# Pretreatment loss to follow-up of tuberculosis patients in Chennai, India: a cohort study with implications for health systems strengthening

**DOI:** 10.1186/s12879-018-3039-3

**Published:** 2018-03-27

**Authors:** Beena E. Thomas, Ramnath Subbaraman, Senthil Sellappan, Chandra Suresh, J. Lavanya, Savari Lincy, Agnes Lawrence Raja, B. Javeed, S. Kokila, S. Arumugam, Soumya Swaminathan, Kenneth H. Mayer

**Affiliations:** 1 Department of Social and Behavioral Research, National Institute for Research in Tuberculosis, No. 1, Mayor Sathiyamoorthy Road, Chetpet, Chennai, 600031 India; 2 Nutrition Infection Unit, Department of Public Health and Community Medicine, Tufts University School of Medicine, 136 Harrison Ave., Boston, 02111 USA; 3 Division of Geographic Medicine and Infectious Diseases, Tufts Medical Center, 260 Tremont St., Boston, 02111 USA; 4 District Tuberculosis Office, No. 26 Pulianthope High Road, Pulianthope, Chennai, 600012 India; 5 World Health Organization Headquarters, Avenue Appia 20, 1202 Geneva, Switzerland; 6 Division of Infectious Diseases, Beth Israel Deaconess Medical Center and Harvard Medical School, 110 Francis St., Boston, 02215 USA; 7 The Fenway Institute, 1340 Boylston St, 8th floor, Boston, MA 02215 USA

**Keywords:** Tuberculosis, India, Cascade of care, Quality of care, Linkage to care, Initial default, Pretreatment loss to follow-up, Implementation science, Health systems research, Operations research

## Abstract

**Background:**

Pretreatment loss to follow-up (PTLFU) is a barrier to tuberculosis (TB) control in India’s Revised National TB Control Programme (RNTCP). PTLFU studies have not been conducted in India’s mega-cities, where patient mobility may complicate linkage to care.

**Methods:**

We collected data from patient registries for May 2015 from 22 RNTCP designated microscopy centers (DMCs) in Chennai and audited addresses and phone numbers for patients evaluated for suspected TB to understand how missing contact information may contribute to PTLFU. From November 2015 to June 2016, we audited one month of records from each of these 22 DMCs and tracked newly diagnosed smear-positive patients using RNTCP records, phone calls, and home visits. We defined PTLFU cases as including: (1) patients who did not start TB therapy within 14 days and (2) patients who started TB therapy but were lost to follow-up or died before official RNTCP registration. We used multivariate logistic regression to identify factors associated with PTLFU.

**Results:**

In the audit of May 2015 DMC registries, out of 3696 patients evaluated for TB, 1273 (34.4%) had addresses and phone numbers that were illegible or missing. Out of 344 smear-positive patients tracked from November 2015 to June 2016, 40 (11.6%) did not start TB therapy within 14 days and 36 (10.5%) started therapy but were lost to follow-up or died before official RNTCP registration, for an overall PTLFU rate of 22.1% (95%CI: 17.8%—26.4%). Of all PTLFU patients, 55 (72.4%) were lost to follow-up and 21 (27.6%) died before starting treatment or before RNTCP registration. In the regression analysis, age > 50 years (OR 2.9, 95%CI 1.4—6.5), history of prior TB (OR 3.9, 95%CI 2.2—7.1), evaluation at a high patient volume DMC (OR 3.2, 95% CI 1.7—6.3), and absence of legible patient contact information (OR 4.5, 95%CI 1.3—15.1) were significantly associated with PTLFU.

**Conclusions:**

In an Indian mega-city, we found a high PTLFU rate, especially in patients with a prior TB history, who are at greater risk for having drug-resistance. Enhancing quality of care and health system transparency is critical for improving linkage of newly diagnosed patients to TB care in urban India.

**Electronic supplementary material:**

The online version of this article (10.1186/s12879-018-3039-3) contains supplementary material, which is available to authorized users.

## Background

India has the highest burden of tuberculosis (TB) patients globally [1], about one-third to half of whom are treated in the Government of India’s Revised National TB Control Programme (RNTCP) [2–4]. A recent national-level analysis suggests that losses of TB patients along multiple steps of the cascade of care may substantially undermine care delivery in the RNTCP [3]. Pretreatment loss to follow-up (PTLFU)—the loss of patients between diagnosis with TB and registration in treatment—is a critical point of attrition in the cascade [3].

Data on PTLFU in India are most robust for smear-positive TB patients. Figures from the RNTCP’s annual reports suggest that more than 135,000 smear-positive patients, or 14.6%, were lost to follow-up prior to starting on TB treatment (as assessed by official registration in the RNTCP) in 2013 [3]. Based on this estimate, more smear-positive patients were lost due to PTLFU than the number who were lost to follow-up, died, or failed treatment after starting their course of TB therapy [3]. TB patients who are lost to follow-up before starting therapy are infectious and have high mortality rates [5, 6]. Also, in most Indian PTLFU studies, a considerable proportion of patients (17%—51%) were untrackable by researchers due to missing or illegible patient phone number and address information in RNTCP registries [7–10].

PTLFU may be especially challenging in India’s mega-cities, because patient mobility may complicate linkage of diagnosed patients to care [11]. In Chennai, India’s fourth most populous city, at least 17% of smear-positive patients diagnosed in the RNTCP have home addresses located outside of the city, mostly in rural districts in Tamil Nadu state [11]. Large cities may also pose challenges for patient retention due to high substance use rates and slum populations with poor access to health services [12, 13].

In this paper, we estimate the prevalence of PTLFU in Chennai and use regression analysis to identify patient- and health system-related factors associated with PTLFU. We also evaluate the role that missing patient contact information may play in contributing to PTLFU through an audit of data from written registries of patients being evaluated for suspected TB at RNTCP microscopy centers. We present an analysis of reasons for PTLFU based on in-depth qualitative interviews with “lost” patients and healthcare providers in a forthcoming companion manuscript.

## Methods

### Study setting

Chennai has a population of about 8.7 million people. The TB prevalence in the city’s general population is about 349 per 100,000 people [14]. RNTCP designated microscopy centers (DMCs) are the primary sites for evaluating TB patients in Chennai, and 63,000 to 67,000 patients are screened for TB annually using sputum microscopy [15, 16]. While Chennai had 54 DMCs in 2014, a recent study highlighted that about 90% of all smear-positive TB patients were diagnosed at just 22 of these DMCs [11]. To make the current study feasible, we evaluated PTLFU at these 22 DMCs that account for the vast majority of the city’s smear-positive diagnoses (see Additional file 1: Table S1 for a list of these DMCs).

Out of these 22 DMCs, four are located either in large tertiary hospitals or in specialized TB facilities: Chennai General Hospital (also known as Madras Medical College), Government Stanley Hospital, the Institute of Thoracic Medicine, and Government Thiruvatteeswarar Hospital of Thoracic Medicine (also known as Otteri TB Hospital). These four DMCs collectively diagnose more than half of all smear-positive TB patients in Chennai, and we will refer to these four DMCs as the “high-volume DMCs.” The remaining 18 DMCs in this study are mostly located in primary or secondary health centers, and we will refer to these 18 DMCs as the “moderate- or low-volume DMCs.”

### Evaluating the quality of patient contact information in DMC registries

Prior studies of PTLFU in India found that many “lost” patients were untrackable by healthcare providers and researchers due to missing or illegible patient phone number and address information in RNTCP registries [7–10]. To better understand this problem in Chennai, we copied and entered into a REDCap database information from patient registries for the month of May 2015 from the 22 DMCs, including all patients with suspected TB who were evaluated with sputum microscopy (“chest symptomatics”) and those diagnosed with smear-positive TB.

Patient phone numbers were coded as being “complete and legible,” “illegible,” or “missing”. Each element of every patient address (i.e., house/flat number, street name, neighborhood, and city/town name) was coded as being “complete and legible,” “illegible,” or “missing.” REDCap (Research Electronic Data Capture) is a web-based application that provides secure data capture with validated data entry, tracking of data manipulation, and automated export procedures for common statistical software packages.

We classified addresses as being “trackable” if every element required to find a patient’s home (i.e., house/flat number, street name, neighborhood, and city/town name) had been recorded legibly. We then classified patients by the likelihood that they could be tracked successfully based on the information in the DMC registry. Patients were classified as “probably trackable” if both a legible phone number and a trackable address were available; “possibly trackable” if only one of these pieces of information was available; and “untrackable” if neither a legible phone number nor a trackable address was available, such that RNTCP staff would have no information with which to find patients who do not return to follow-up on sputum test results.

### Case definitions for pretreatment loss to follow-up

**Fig. 1:**
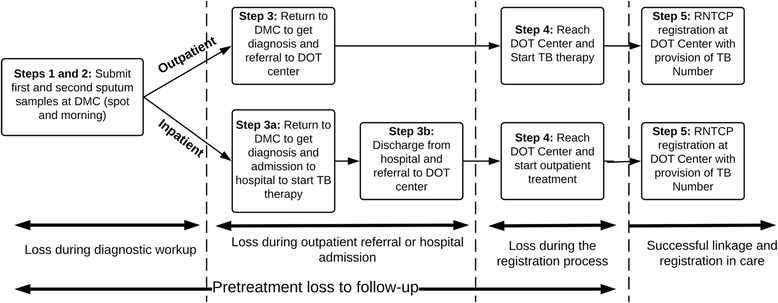
The process of diagnosis and linkage to care for smear-positive tuberculosis patients referred for initial treatment in the outpatient or inpatient setting. TB = tuberculosis; DMC = designated microscopy center; DOT = directly observed therapy; RNTCP = Revised National Tuberculosis Control Programme

Linkage of newly diagnosed smear-positive patients to care in the RNTCP is not a single event but rather a multistep process, which varies based on whether a patient is initially referred for treatment to the outpatient or inpatient setting (Fig. 1). Patients first submit two sputum samples—a “spot” sample upon arrival and a “morning” sample the next day. He or she then returns to the DMC a third time to find out the sputum test result.

Those initially treated as outpatients get referred to a DOT center close to their homes. Upon arrival at the DOT center, the patient is usually started on TB treatment. RNTCP registration with provision of an official “TB Number” may happen simultaneously with treatment initiation; however, there is sometimes delay in registration until RNTCP staff can conduct a home visit to confirm the patient’s address or until a Senior Treatment Supervisor (STS), who often manages multiple DOT centers, can reach the center to complete the paperwork (Fig. 1).

Linkage to care is a more complex process for patients initially referred for a brief inpatient admission, where they usually start TB treatment but are not registered in the RNTCP (Fig. 1). After discharge from the hospital, they are referred to an outpatient DOT center, where TB treatment is continued. RNTCP registration may again be delayed at the DOT center pending a home visit or visit of a STS. For both outpatient and inpatient referrals, there are multiple steps at which patients may be lost to follow-up (e.g., during diagnostic workup, referral, hospital admission, or RNTCP registration) (Fig. 1).

For all patients, we define successful linkage to care as consisting of RNTCP registration, as confirmed by provision of an official TB Number. We use RNTCP registration as the primary outcome (rather than treatment initiation) for a few reasons. First, registration is supposed to be required for all patients started on TB treatment in the RNTCP. TB Numbers therefore remain the simplest way of confirming treatment initiation using RNTCP records. Most prior studies of PTLFU in India used RNTCP registration, as determined by an audit of TB Numbers, to confirm treatment initiation [8–10, 17–21]. Once a patient is registered, his or her treatment outcome is reported as part of local and national TB statistics; healthcare providers may therefore be more motivated to ensure engagement of patients in TB care after RNTCP registration.

Since TB treatment initiation and RNTCP registration do not always happen simultaneously, we define PTLFU as including two types of patients: (1) patients ≥18 years of age diagnosed with smear-positive TB in the RNTCP who did not start therapy at a DOT center or in the private sector within 14 days of the first positive sputum sample; and (2) patients ≥18 years of age diagnosed with smear-positive TB who started therapy in the RNTCP but were lost to follow-up or died before RNTCP registration. By defining PTLFU as including these two different groups, our study also sheds light on the impact of delays in RNTCP registration on PTLFU. We further classified each PTLFU case into the following outcomes: (1) alive and trackable; (2) died; and (3) untrackable (described further below).

We also classified each PTLFU patient based on the point in the diagnostic, referral, hospital admission, and RNTCP registration process at which he or she was lost to follow-up (Fig. 1); however, we present those findings as part of a forthcoming companion manuscript analyzing the qualitative study findings.

### Data collection for evaluation of pretreatment loss to follow-up

Between October 2015 to June 2016, we tracked all newly diagnosed smear-positive TB patients ≥18 years of age for one four-week time period at each of the 22 DMCs. To facilitate study feasibility, patient tracking was conducted at 5 or 6 DMCs during each four-week time period, until all 22 DMCs had been covered. Prior to conducting the study, we estimated that collecting data on all smear-positive patients for one four-week time period at each of the 22 DMCs would yield a sample of 300 to 400 patients. Based on a systematic review of prior studies [3], we anticipated that we would find a PTLFU rate ranging from 10% to 20% in Chennai, and the sample size would provide adequate power to estimate the PTLFU rate within a confidence interval of +/− 5%.

**Fig. 2:**
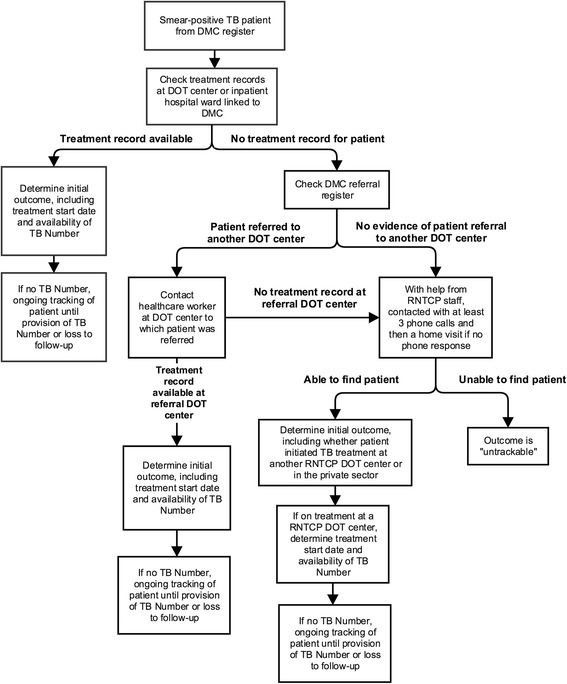
Protocol for determining study outcomes for smear-positive tuberculosis patients tracked by the field research team. TB = tuberculosis; DMC = designated microscopy center; DOT = directly observed therapy; RNTCP = Revised National Tuberculosis Control Programme

We followed a pre-defined protocol to track patients starting no earlier than 14 days and no later than 21 days after the first positive sputum smear (Fig. 2). We checked whether the patient had started therapy at the DOT center linked to the DMC or at other DOT centers to which the patient may have been referred. If no treatment record was found, field researchers worked with RNTCP staff to attempt to contact the patient with at least three phone calls, followed by a home visit for patients not reachable by phone. Patients were classified as being “untrackable” if the study team was unable to find them after these phone calls and home visits.

To understand reasons for PTLFU, in-depth qualitative interviews were conducted with PTLFU patients who were trackable or with family members of PTLFU patients who had died. The interview methods and the qualitative findings are reported in a separate forthcoming manuscript.

For patients who started TB treatment within 14 days, we checked whether they had been assigned TB Numbers, which confirm RNTCP registration (Fig. 2). We continued to track patients who had not been assigned a TB Number within 14 days until they received TB Numbers; however, in some cases, these patients were lost to follow-up or died before being assigned TB Numbers. These patients who were lost to follow-up or died before RNTCP registration were tracked, and qualitative interviews were conducted with trackable patients or with the families of patients who had died.

### Retrieval of “lost” patients by the study team

The study team worked with RNTCP staff to re-engage into TB care PTLFU patients who were alive and trackable. We carefully documented the outcome of these efforts to retrieve patients to provide insight into the potential yield that intensive patient tracking initiatives might have on reducing PTLFU in the future. The study team, which consisted of trained social workers, provided basic TB knowledge and treatment counseling to all PTLFU patients after finishing the qualitative interviews. These PTLFU patients were then followed up to determine whether they started TB treatment or did not re-engage in care.

### Analysis of quantitative data

To identify predictors associated with PTLFU, we conducted two different logistic regression analyses, based on the two groups of patients included in our case definition of PTLFU. First, we built a multivariate model with the dependent variable being failure to start therapy within 14 days of the first positive sputum sample. Second, we built a multivariate model with the dependent variable being the overall definition of PTLFU, which includes patients who did not start therapy and those who started therapy but were lost to follow-up or died before RNTCP registration.

These two analyses provide different insights into linkage to care. While first analysis identifies factors associated with not starting TB therapy, the second analysis identifies factors that may affect the entire process of linkage to care, including RNTCP registration (Fig. 1).

We used JMP Pro 12 to build the regression model including the following covariates: (1) gender; (2) age; (3) history of prior TB treatment; (4) ease of patient trackability (classified as probably trackable, possibly trackable, or untrackable as described above); (5) whether the patient’s home is located inside or outside of Chennai city; and (6) whether the patient was diagnosed at a high patient volume DMC or a lower-volume DMC in the city. History of prior TB treatment was mostly based on information captured in the DMC and referral registries. However, we also classified patients started on Category 2 or multidrug-resistant (MDR) TB therapy as having a prior history of TB, since current RNTCP guidelines only recommend Category 2 therapy and screening for drug-resistance for patients with a prior history of TB treatment [22]. The STROBE checklist for reporting of cohort studies is included as Additional file 2.

## Results

### “Trackability” based on audit of patient contact information

Based on the audit of May 2015 DMC registries, out of 3696 chest symptomatics who submitted sputum samples for evaluation, 2231 (60.4%) had legible phone numbers recorded, while 606 (16.4%) had complete and legible addresses recorded (Table 1). Out of 423 patients diagnosed with smear-positive TB, 316 (74.7%) had legible phone numbers recorded, while 56 (13.2%) had complete and legible addresses recorded. Only 414 (11.2%) chest symptomatics and 43 (10.2%) diagnosed smear-positive patients were “probably trackable” because both a phone number and legible address information were recorded, while 1273 (34.4%) chest symptomatics and 94 (22.2%) diagnosed smear-positive patients were “untrackable” because neither a phone number nor a legible address was recorded.

**Table 1:** Quality of patient address and phone number information in designated microscopy center (DMC) registries in Chennai and ease of patient trackability

	Chest symptomatics (*n* = 3696)	Diagnosed smear-positive patients (*n* = 423)
	N (%)	N (%)
Phone number listed in DMC registry		
Yes	2231 (60.4)	316 (74.7)
No	1454 (39.3)	104 (24.6)
Illegible	11 (0.3)	3 (0.7)
Address listed in DMC registry		
Trackable	606 (16.4)	56 (13.2)
Incomplete or illegible	3046 (83.4)	367 (86.8)
Missing	44 (1.2)	0 (0.0)
Ease of “trackability”		
Probably trackable^a^	414 (11.2)	43 (10.2)
Possibly trackable^a^	2009 (54.4)	286 (67.6)
Untrackable^a^	1273 (34.4)	94 (22.2)

^a^Patients were “probably trackable” if a legible phone number and trackable address were available; “possibly trackable” if only one of the two was available; and “untrackable” if neither a legible phone number nor trackable address was available

### Prevalence of, and risk factors for, pretreatment loss to follow-up

**Fig. 3:**
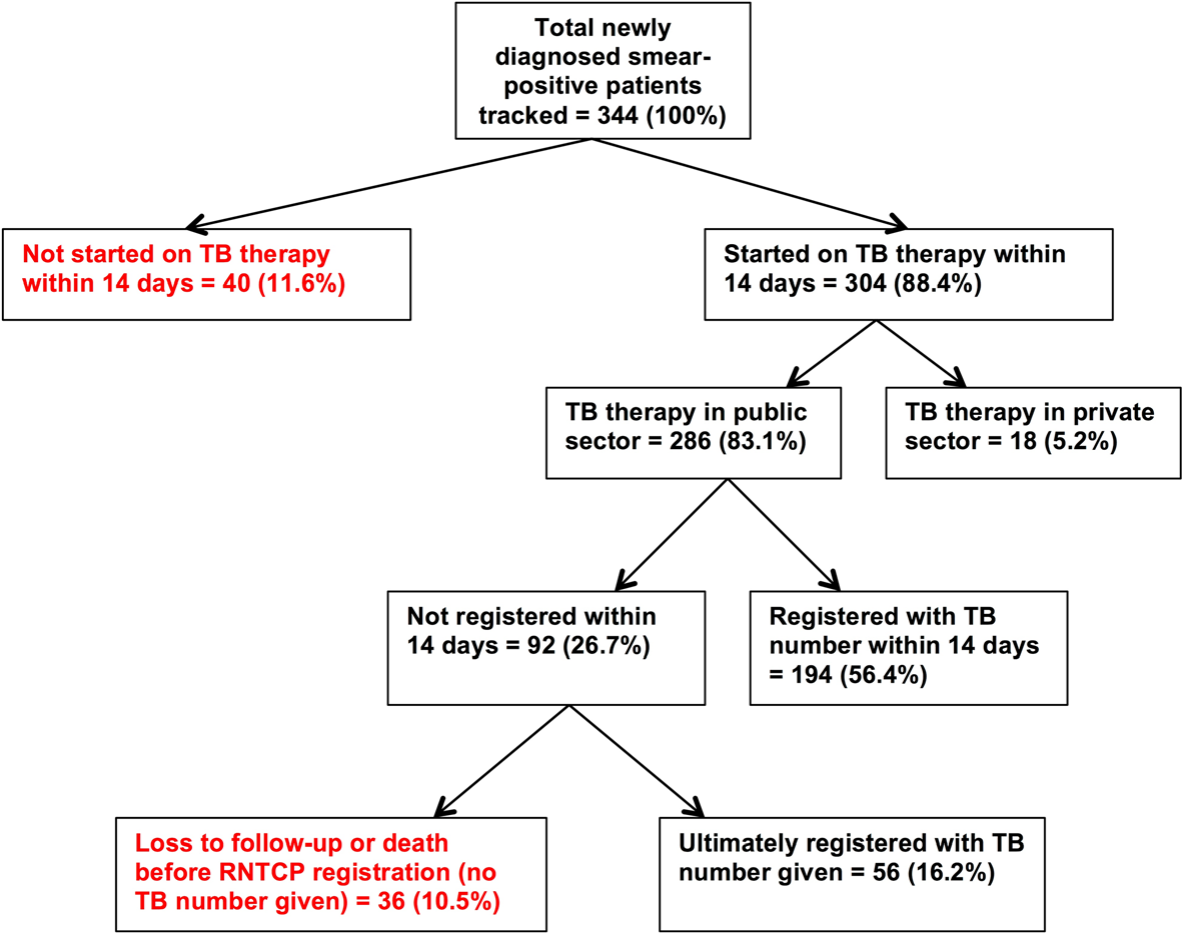
Pretreatment loss to follow-up outcomes for 344 tuberculosis patients tracked in Chennai’s government TB program, including patients who failed to start therapy within two weeks and patients who did not get registered in the RNTCP. All percentages are based on the denominator of 344 smear-positive patients tracked. TB = tuberculosis; RNTCP = Revised National TB Control Programme

Out of 344 smear-positive patients tracked from November 2015 to June 2016, we found 76 cases of PTLFU (22.1%; 95%CI: 17.8%—26.4%). Of these PTLFU cases, 40 patients (11.6%; 95%CI: 8.3%—14.9%) did not start TB therapy within 14 days of the first positive sputum sample and 36 patients (10.5%; 95%CI: 7.7%—14.2%) started therapy but died or were lost to follow-up before RNTCP registration (Fig. 3).

Table 2 presents findings of the multivariate logistic regression model evaluating factors associated with failure to start TB therapy within 14 days of diagnosis. Age > 50 years, having a home address located outside of Chennai city, and lacking both phone and address information in the DMC registry (i.e., being “untrackable”) are significantly associated with failure to start therapy. Notably, being diagnosed at a high patient volume DMC (as opposed to a moderate- or low-volume DMC) is close to meeting the threshold for statistical significance (*p* = 0.07).

**Table 2:** Factors associated with failure of smear-positive tuberculosis patients to start therapy within 14 days of diagnosis in Chennai, India, in a multivariate logistic regression analysis

	Descriptive statistics		Regression model		
	Proportion of sample (*N* = 344)	Proportion who did not start TB treatment	Univariate Findings	Multivariate findings (N = 344)	*p*-value
	*N(%)*	*N(%)*	*Odds Ratio (p-value)*	*Odds Ratio* *(CI)*	
Gender					
Male	280 (81.4)	35 (12.5)	–	–	
Female	64 (18.6)	5 (7.8)	0.59 (0.27)	0.82 (0.26–2.21)	0.70
Age					
18–35	90 (26.2)	6 (6.7)	–	–	
36–50	141 (41.0)	13 (9.2)	1.42 (0.48)	0.99 (0.35–3.09)	0.99
51+	113 (32.9)	21 (18.6)	3.19 (0.01)*	2.70 (1.06–7.84)	0.04*
Patient from inside or outside of Chennai					
Inside Chennai	281 (81.7)	25 (8.9)	–	–	
Outside Chennai	63 (18.3)	15 (23.8)	3.2 (0.002)*	3.01 (1.37–6.52)	0.007*
Ease of trackability based on contact information					
Probably trackable	201 (58.4)	17 (8.5)	–	–	
Possibly trackable	128 (37.2)	19 (14.8)	1.88 (0.07)	1.45 (0.68–3.08)	0.33
Untrackable	15 (4.4)	4 (26.7)	3.94 (0.049)*	4.53 (1.08–16.52)	0.04*
Prior TB treatment history					
No prior TB treatment	247 (71.8)	25 (10.1)	–	–	
Prior TB treatment	97 (28.2)	15 (15.5)	1.62 (0.16)	1.79 (0.83–3.77)	0.13
Site of initial microscopy test					
Moderate or low patient volume microscopy center	133 (38.7)	10 (7.5)	–	–	
High patient volume microscopy center	211 (61.3)	30 (14.2)	2.04 (0.053)	2.02 (0.94–4.68)	0.07

*indicates a statistically significant finding at the 5% level

Table 3 presents findings of the multivariate logistic regression model evaluating factors associated with PTLFU, using the overall case definition that includes patients who failed to start TB therapy within 14 days and those who started therapy but were lost to follow-up or died before RNTCP registration. Age > 50 years, having a history of prior TB treatment, lacking phone and address information in the DMC registry (i.e., being “untrackable”), and being diagnosed at a high patient volume DMC are significantly associated with PTLFU.

**Table 3:** Factors associated with pretreatment loss to follow-up (PTLFU) of smear-positive tuberculosis patients in Chennai, India, in a multivariate logistic regression analysis

	Descriptive statistics		Regression model		
	Proportion of sample (N = 344)	Proportion with PTLFU	Univariate Findings	Multivariate findings (N = 344)	*p*-value
	*N(%)*	*N(%)*	*Odds Ratio (p-value)*	*Odds Ratio* *(CI)*	
Gender					
Male	280 (81.4)	63 (22.5)	–	–	
Female	64 (18.6)	13 (20.3)	0.88 (0.70)	1.33 (0.59—2.88)	0.49
Age					
18–35	90 (26.2)	13 (14.4)	–	–	
36–50	141 (41.0)	26 (18.4)	1.34 (0.43)	0.97 (0.44—2.20)	0.93
51+	113 (32.8)	37 (32.7)	2.88 (0.002)	2.94 (1.40—6.49)	0.004*
Patient from inside or outside of Chennai					
Inside Chennai	281 (81.7)	60 (21.4)	–	–	
Outside Chennai	63 (18.3)	16 (25.4)	1.25 (0.49)	1.18 (0.56—2.38)	0.66
Trackability based on phone number and address information					
Probably trackable	201 (58.4)	35 (17.4)	–	–	
Possibly trackable	128 (37.2)	35 (27.3)	1.78 (0.03)	1.66 (0.91—3.05)	0.10
Untrackable	15 (4.4)	6 (40.0)	3.16 (0.05)	4.49 (1.29—15.06)	0.02*
Prior TB treatment history					
No prior TB treatment	247 (71.8)	38 (15.4)	–	–	
Prior TB treatment	97 (28.2)	38 (39.2)	3.54 (< 0.001)	3.88 (2.15—7.09)	< 0.0001*
Site of initial microscopy test					
Moderate or low patient volume microscopy center	133 (38.7)	16 (12.0)	–	–	
High patient volume microscopy center	211 (61.3)	60 (28.4)	2.91 (0.0002)	3.18 (1.69—6.32)	0.0002*

*indicates a statistically significant finding at the 5% level

### Outcomes of pretreatment loss to follow-up cases

Table 4 describes the outcomes of patients who did not start TB therapy within 14 days, who started therapy but were lost to follow-up or died before RNTCP registration, and who suffered from PTLFU for either reason.

**Table 4:** Outcomes of pretreatment lost to follow-up patients

Patients who did not start TB therapy within 14 days of diagnosis (*n* = 40)	*N (%)*
Alive and trackable by study team	21 (52.5)
Not trackable by study team	17 (42.5)
Died before starting therapy	2 (5.0)
Patients who started TB therapy but who were lost to follow-up or died before RNTCP registration (n = 36)	
Alive and trackable by study team	7 (19.4)
Not trackable by study team	10 (27.8)
Died after starting therapy but before RNTCP registration	19 (52.8)
All PTLFU patients (*n* = 76)	
Alive and trackable by study team	28 (36.8)
Not trackable by study team	27 (35.5)
Died before starting therapy or before RNTCP registration	21 (27.6)

Of the overall sample of 76 PTLFU patients, 28 (36.8%) were tracked and found alive by the study team; 27 (35.5%) were lost to follow-up but not trackable despite the study team’s best efforts; and 21 (27.6%) had died. Notably, of PTLFU patients who had died, only 2 died before starting TB therapy, and 19 died after starting TB therapy but before official RNTCP registration.

### Retrieval of “lost” patients by the study team

Out of the 28 PTLFU patients who were successfully tracked and found to be alive, the study team was able to successfully retrieve 19 (67.9%) patients and get them started on TB therapy. Three (10.7%) patients had not started TB therapy within 14 days but had re-engaged in TB care themselves before being contacted by the study team, and 6 (21.4%) patients had ongoing non-engagement in TB care, despite the study team’s best efforts.

## Discussion

In this study of PTFLU in an India mega-city, we estimate that 22% of smear-positive TB patients fail to start therapy, are lost to follow-up, or die before RNTCP registration. This PTLFU rate is higher than national estimates of 16% based on a meta-analysis of local Indian studies and 14.6% for the year 2013 based on estimates from RNTCP reports [3]. Given this relatively high PTLFU rate, future studies should be conducted in other major Indian cities to assess whether poor linkage to care is a problem for urban TB control nationally.

A recent paper highlighted an “urban registration gap” in many large Indian cities, in which more smear-positive patients are diagnosed every year than the number who are registered in TB therapy within those cities per RNTCP reports [11]. For example, in Chennai, 6135 smear-positive patients were diagnosed while only 3148 were registered in treatment in 2013, for a gap of 49% [15]. While some of this gap is accounted for by patients who receive TB therapy outside of Chennai [11], this current study suggests that PTLFU may explain a considerable proportion of the remaining urban registration gap. Our findings may have implications for other major Indian cities that also have unexplained urban registration gaps.

PTLFU patients have historically not been reported in official statistics by TB programs. Our estimates suggest that their inclusion in official RNTCP statistics would have a considerable impact on the program’s treatment outcomes. For example, in the RNTCP’s 2014 report, new smear-positive and retreatment smear-positive patients in Chennai were reported as having treatment completion rates of 86% and 69%, respectively. Extrapolating from our study findings, if PTLFU cases were included in patient outcomes, we estimate that treatment completion rates in Chennai would be revised downward considerably to 73% and 42% for new smear-positive and retreatment smear-positive patients, respectively.

Our study shows that PTLFU is shaped by a multistep process of linkage to care. Most prior PTLFU studies have assumed that RNTCP registration (i.e., provision of a TB Number) is a reasonable surrogate for TB treatment initiation [8–10, 17–21]. However, we found that, despite starting TB treatment, many patients were lost to follow-up before official registration. Our findings therefore suggest that delays in RNTCP registration contribute considerably to the overall PTLFU rate. The RNTCP recently set a goal of officially registering all TB patients at the time of diagnosis, rather than after they reach a DOT center and start therapy [22, 23]. While implementation of this “registration at diagnosis” policy may result in an apparent worsening of treatment outcomes in the short-term, if stringently enforced, it could improve health system transparency and motivate improvements in linkage to care.

In the regression analyses, we identified health system- and patient-related factors independently associated with PTLFU. Poor quality of patient contact information in DMC registries is a major factor associated with PTLFU. Missing or incomplete information could partly be related to lack of phone access or lack of a stable home address on the part of patients. However, our analysis of DMC registries for the month of May 2015 suggests that recording error—illegible handwriting and addresses that are incompletely recorded (e.g., missing the house number)—is the primary problem compromising > 80% of addresses. Notably, studies conducted in Chennai nearly four decades ago similarly found poor quality of patient address information to be a problem compromising TB care delivery, though only one-fifth of recorded addresses had critical deficiencies at that time [24, 25].

Patients who visited high-volume DMCs were more likely to experience PTLFU. Chennai’s high-volume DMCs are located in well-known hospitals or TB specialty facilities that draw patients living throughout the city and Tamil Nadu state [11]. After diagnosis, most are referred back to the DOT centers closest to their homes to start therapy in the RNTCP’s facility-based DOT model. This circuitous referral process might increase the risk of PTLFU. In contrast, patients visiting moderate- or low-volume DMCs, which are mostly located in primary or secondary health centers closer to patients’ homes, may be more likely to start TB therapy in that same center or at a DOT center relatively nearby, which might facilitate linkage to care. In addition, patients visiting moderate- or low-volume DMCs may be more likely to have positive personal interactions with healthcare providers, due to lower patient volume at these facilities [11].

Having a prior history of TB treatment is one of the most concerning factors associated with PTLFU from a public health perspective, because these patients are at higher risk for having and transmitting drug-resistant TB [26, 27]. Nearly 40% of patients with a history of prior TB treatment experienced PTLFU. Prior TB treatment was a risk factor for overall PTLFU but not for failure to start TB therapy, suggesting that these patients are at particularly high risk for loss to follow-up prior to RNTCP registration.

Individuals > 50 years of age were also at greater risk for PTLFU; so older patients should be a focus of interventions aiming to reduce PTLFU. Patients with an address outside of Chennai were at higher risk for not starting TB therapy. Challenges in coordination of the referral process between high-volume city DMCs and rural DOT centers may prevent or delay TB treatment initiation for these patients [11].

Some of the risk factors for PTLFU that we identify in this study—including a history of prior TB treatment and initial diagnosis at high patient volume facilities—are novel contributions to the Indian literature on PTLFU. Our finding that patients with addresses outside of Chennai are less likely to initiate TB therapy affirms findings from prior studies, which found that greater distance of patients’ homes from DMCs and rural-to-urban migration increased the risk of PTLFU [10, 21, 28]. Other factors contributing to PTLFU identified in prior Indian studies include dissatisfaction with government health services [7, 10], employment-related barriers [7, 21], alcohol use disorder [29], and TB-related stigma [29].

Partly as a result of the poor quality of patient contact information, about one-third of PTLFU patients were not trackable by the study team, which is similar to findings from prior Indian studies [7–10]. This suggests that illegible or incomplete patient information in DMC registries may be a national problem. About one-third of PTLFU patients were tracked and found to be alive by the study team. The study team was able to re-engage more than two-thirds of these patients in TB care, with the help of RNTCP staff. This finding suggests that a proactive patient tracking intervention, especially with a dedicated patient retention team, may be an effective strategy for reducing PTLFU in the RNTCP.

This study has a few limitations. First, to ensure study feasibility, we did not sample patients from the 32 lowest-volume DMCs that collectively diagnose about 10% of the city’s smear-positive patients. If the rates of linkage to care at these facilities are similar to those at other primary and secondary DMCs in Chennai, it is possible that we slightly overestimate the overall PTLFU rate. Second, while we tried to minimize the number of untrackable patients by implementing a rigorous protocol, many patients were not trackable despite the study team’s best efforts. Some of these patients could have potentially engaged in TB care at other sites inside or outside of Chennai, leading to overestimation of the PTLFU rate. Alternatively, if some of these patients died, it is possible that we underestimate mortality for PTLFU cases.

Finally, we were not able to include HIV co-infection as a covariate in our analysis, because newly diagnosed TB patients are usually referred to separate voluntary counseling and testing centers for HIV testing after TB diagnosis. As a result, HIV status was only known for patients who reported a pre-existing HIV diagnosis at the time of DMC evaluation. Similarly, our data on TB drug-resistance for most patients were limited. At the time of our study, all smear-positive patients with a history of prior TB treatment were supposed to undergo screening for drug-resistance with a line probe assay (LPA). However, since LPA evaluation is conducted on fresh sputum samples collected after initial TB diagnosis, drug-resistance testing was not conducted upfront for most PTLFU patients with a prior TB treatment history and delivery of LPA results was slow even for patients who were successfully linked to care. As such, we were not able to include drug-resistance as a covariate in our analysis, though at least one of the PTLFU patients in this cohort had confirmed MDR TB.

## Conclusions

In India’s fourth most populous city, we found a high PTLFU rate. Older patients and patients with a history of prior TB treatment should be a major focus of interventions to reduce PTLFU. We further explore patient-related reasons for PTLFU in a forthcoming companion manuscript analyzing qualitative data from this study.

Health system challenges were some of the most prominent factors contributing to PTLFU. Improving the quality of patient contact information recorded at the time of sputum evaluation (potentially through regular audits of records with performance feedback) [30], proactive patient tracking by a healthcare worker team dedicated to patient retention, and rigorous implementation of a “registration at diagnosis” policy may help to reduce PTLFU and improve linkage to care. High-volume DMCs in cities should be priority sites for these health system-strengthening interventions, with a goal of improving coordination with the rural DOT centers where many out-of-city patients may start TB therapy.

## Additional files

Additional file 1: **Table S1** Revised National Tuberculosis Control Programme (RNTCP) designated microscopy centers (DMCs) in Chennai included in this study. (DOCX 127 kb)

Additional file 2: STROBE checklist for cohort studies. (DOC 85 kb)

## Abbreviations

CI: Confidence interval
DMC: Designated microscopy center
DOT: Directly observed therapy
LPA: Line probe assay
OR: Odds ratio
PTLFU: Pretreatment loss to follow-up
RNTCP: Revised National Tuberculosis Control Programme
STS: Senior Treatment Supervisor
TB: Tuberculosis
