# Supplementary material for: Pretreatment loss to follow-up of tuberculosis patients in Chennai, India: a cohort study with implications for health systems strengthening
Source: BMC Infect Dis. 2018 Mar 27;18:142. doi: 10.1186/s12879-018-3039-3 (PMC5872574; doi:10.1186/s12879-018-3039-3)
Supplement: Supplementary file 1 — Table S1 Revised National Tuberculosis Control Programme (RNTCP) designated microscopy centers (DMCs) in Chennai included in this study. (DOCX 127 kb) [file 12879_2018_3039_MOESM1_ESM.docx]

**Additional file 1. Revised National Tuberculosis Control Programme (RNTCP) designated microscopy centers (DMCs) in Chennai included in this study**

Table S1. Distribution of chest symptomatics and diagnosed smear-positive tuberculosis patients at the 22 highest-volume designated microscopy centers in Chennai, India, 2014

| Name of facility and months patient tracking was conducted | TB suspects evaluated  (n=69,200)  N (%)^a^ | Smear-positive TB cases diagnosed  (n=6,135)  N (%)^b^ |
| --- | --- | --- |
| Otteri TB Hospital (November—December 2015) | 6,381 (9.2) | 1317 (21.5) |
| Institute of Thoracic Med. (December 2015—January 2016) | 9,478 (13.7) | 798 (13.0) |
| Madras Medical College (June—July 2016) | 7,132 (10.3) | 779 (12.7) |
| Stanley Hospital (October—November 2015) | 4,788 (6.9) | 604 (9.8) |
| Royapettah Hospital (June—July 2016) | 3,133 (4.5) | 306 (5.0) |
| GPH, KK Nagar (December 2015—January 2016) | 1,778 (2.6) | 231 (3.8) |
| Sri Ramachandra Medical College (December 2015—January 2016) | 1,892 (2.7) | 196 (3.2) |
| Pulianthope TB Clinic (October—November 2015) | 2,274 (3.3) | 187 (3.0) |
| Kilpauk Medical College (December 2015—January 2016) | 4,727 (6.8) | 178 (2.9) |
| Communicable Disease Hospital (October—November 2015) | 1,618 (2.3) | 119 (1.9) |
| Thiruvanmiyur UPHC (June—July 2016) | 990 (1.4) | 103 (1.7) |
| Basin Bridge UPHC (October—November 2015) | 1,131 (1.6) | 97 (1.6) |
| GPH, Tondiarpet (June—July 2016) | 1,185 (1.7) | 92 (1.5) |
| GPH, Anna Nagar (October—November 2015) | 1,356 (2.0) | 70 (1.1) |
| Thiruvetriyur (November—December 2015) | 836 (1.2) | 69 (1.1) |
| Saidapet General Hospital (December 2015—January 2016) | 1,099 (1.6) | 67 (1.1) |
| Kodambakkam UPHC (December 2015—January 2016) | 921 (1.3) | 62 (1.0) |
| Thanthai Periyar UPHC (November—December 2015) | 842 (1.2) | 59 (1.0) |
| ESI Hospital, Ayanavaram (June—July 2016) | 1,511 (2.2) | 58 (0.9) |
| Nungambakkam UPHC (November—December 2015) | 867 (1.3) | 47 (0.8) |
| GPH, Periyar Nagar (November—December 2015) | 676 (1.0 | 45 (0.7) |
| Mylapore UPHC (June—July 2016) | 438 (0.6) | 36 (0.6) |

TB=tuberculosis; GPH=government public hospital; UPHC=universal primary health center.

^a^This percentage represents the number of people with suspected TB evaluated at each facility divided by the total number of 69,200 people with suspected TB evaluated in Chennai in 2014.

^b^This percentage represents the number of smear-positive TB patients diagnosed at each facility divided by the total number of 6,135 smear-positive TB patients diagnosed in Chennai in 2014.
