# Supplementary material for: Pretreatment loss to follow-up of tuberculosis patients in Chennai, India: a cohort study with implications for health systems strengthening
Source: BMC Infect Dis. 2018 Mar 27;18:142. doi: 10.1186/s12879-018-3039-3 (PMC5872574; doi:10.1186/s12879-018-3039-3)
Supplement: Supplementary file 2 — STROBE checklist for cohort studies. (DOC 85 kb) [file 12879_2018_3039_MOESM2_ESM.doc]

**Appendix 2. Strobe checklist for cohort studies**

|  | Item No | Recommendation |
| --- | --- | --- |
| **Title and abstract** | 1 | (*a*) Indicate the study’s design with a commonly used term in the title or the abstract  Page 1: title describes our method as a “cohort study” |
|  |  | (*b*) Provide in the abstract an informative and balanced summary of what was done and what was found  Page 4: abstract describes study sites, patient selection criteria, cases definitions, cohort outcomes, and risk factors associated with cohort outcomes |
| Introduction | | |
| Background/rationale | 2 | Explain the scientific background and rationale for the investigation being reported  Pages 6, 7: the “background” section highlights the importance of pretreatment loss to follow-up for TB control, describes findings from prior studies of this issue in India, and describes our rationale for studying this topic in Chennai, India |
| Objectives | 3 | State specific objectives, including any prespecified hypotheses  Page 7: describes our primary objective of determining the pretreatment loss to follow-up rate for TB patients in Chennai and describes our hypothesis that missing patient contact information may contribute to this problem |
| Methods | | |
| Study design | 4 | Present key elements of study design early in the paper  Pages 8—12: Describe key elements of our study design, including data collection for evaluating patient contact information (page 8), data collection for evaluating pretreatment loss to follow-up (page 11), our protocol for tracking patients (page 11) |
| Setting | 5 | Describe the setting, locations, and relevant dates, including periods of recruitment, exposure, follow-up, and data collection  Pages 7—8: describe the study location/setting in detail  Page 8: describes time period for data collection for evaluating of patient contact information  Page 11 and Supplementary Appendix: describes time period for data collection, patient recruitment and patient tracking for the study of pretreatment loss to follow-up |
| Participants | 6 | (*a*) Give the eligibility criteria, and the sources and methods of selection of participants. Describe methods of follow-up  Page 8: describes eligible patient data for the audit of patient contact information  Page 11: describes criteria for patients eligible for inclusion and tracking in the study of pretreatment loss to follow-up; describes methods for tracking and follow-up of these patients, including contacting healthcare workers, phone calls to patients, and home visits |
|  |  | (*b*) For matched studies, give matching criteria and number of exposed and unexposed  N/A |
| Variables | 7 | Clearly define all outcomes, exposures, predictors, potential confounders, and effect modifiers. Give diagnostic criteria, if applicable  Page 9—11: extensive discussion of case definitions of the outcomes for the cohort study  Page 12—13: description of covariates / predictors included in the analysis |
| Data sources/ measurement | 8* | For each variable of interest, give sources of data and details of methods of assessment (measurement). Describe comparability of assessment methods if there is more than one group  Page 8: describes data sources for the evaluation of patient contact information  Page 11: describes data sources for the outcomes of the pretreatment loss to follow-up study and predictors |
| Bias | 9 | Describe any efforts to address potential sources of bias  Page 11: we describe extensive efforts placed into tracking all patients, to minimize the number of “untrackable” patients |
| Study size | 10 | Explain how the study size was arrived at  Page 11: describes the anticipated sample size, which was calculated to achieve a confidence interval of +/-5% or less for all possible PTLFU rates |
| Quantitative variables | 11 | Explain how quantitative variables were handled in the analyses. If applicable, describe which groupings were chosen and why  Pages 12—13: describe how quantitative variables were handled |
| Statistical methods | 12 | (*a*) Describe all statistical methods, including those used to control for confounding  Page 12: describes methods for the regression analysis |
|  |  | (*b*) Describe any methods used to examine subgroups and interactions  N/A |
|  |  | (*c*) Explain how missing data were addressed  N/A |
|  |  | (*d*) If applicable, explain how loss to follow-up was addressed  Pages 9—12: loss to follow-up a key outcome of the cohort study |
|  |  | (*e*) Describe any sensitivity analyses  N/A |
| Results | | |
| Participants | 13* | (a) Report numbers of individuals at each stage of study—eg numbers potentially eligible, examined for eligibility, confirmed eligible, included in the study, completing follow-up, and analysed  Pages 14—15 and Figure 3: reports that numbers of individuals at each stage of the PTFLU study |
|  |  | (b) Give reasons for non-participation at each stage  Pages 18—19: give outcomes of the pretreatment loss to follow-up patients and predictors of pretreatment loss to follow-up, which essentially provides insights into non-participation |
|  |  | (c) Consider use of a flow diagram  Figure 3 is a flow diagram showing the flow of study participants |
| Descriptive data | 14* | (a) Give characteristics of study participants (eg demographic, clinical, social) and information on exposures and potential confounders  Tables 2 and 3, specifically the columns labelled “proportion of sample” and “proportion who did not start TB treatment” or “proportion with PTLFU” describe the demographic, clinical, and social variables for the cohort |
|  |  | (b) Indicate number of participants with missing data for each variable of interest  There were not missing data for the variables of interest. |
|  |  | (c) Summarise follow-up time (eg, average and total amount)  Page 14: describes study duration; Pages 14—18: describe follow-up time (all patients were tracked as soon as possible starting 14 days after diagnosis) |
| Outcome data | 15* | Report numbers of outcome events or summary measures over time  Pages 18—19 and Figure 3 summarize the numbers of patients with different outcomes |
| Main results | 16 | (*a*) Give unadjusted estimates and, if applicable, confounder-adjusted estimates and their precision (eg, 95% confidence interval). Make clear which confounders were adjusted for and why they were included  Tables 2 and 3 both provide unadjusted (univariate) and adjusted (multivariate) findings for every variable, including 95% confidence intervals for the odds ratios in the multivariate analysis. As noted in the methods and tables, all confounders were adjusted for that were analysed (i.e., there was no stepwise elimination) |
|  |  | (*b*) Report category boundaries when continuous variables were categorized  Tables 2 and 3 describe the breakdown of continuous variables such as age into discrete categories |
|  |  | (*c*) If relevant, consider translating estimates of relative risk into absolute risk for a meaningful time period  N/A |
| Other analyses | 17 | Report other analyses done—eg analyses of subgroups and interactions, and sensitivity analyses  N/A |
| Discussion | | |
| Key results | 18 | Summarise key results with reference to study objectives  Page 20—23: describes results that correspond to key objectives including: (a) PTLFU rate; (b) risk factors associated with PTLFU; (c) quality of patient contact information; and (d) outcomes of PTLFU patients |
| Limitations | 19 | Discuss limitations of the study, taking into account sources of potential bias or imprecision. Discuss both direction and magnitude of any potential bias  Page 23: discuss study limitation, including potential reasons for over- or under-estimation of PTLFU rates |
| Interpretation | 20 | Give a cautious overall interpretation of results considering objectives, limitations, multiplicity of analyses, results from similar studies, and other relevant evidence  Pages 20—23: interprets results in context of prior literature |
| Generalisability | 21 | Discuss the generalisability (external validity) of the study results  Page 20: highlights potential generalizability to other large Indian cities that have unexplained “urban registration gaps”  Page 24: we highlight that other major cities may be priority sites for interventions to address pretreatment loss to follow-up in the conclusion of the paper |
| Other information | | |
| Funding | 22 | Give the source of funding and the role of the funders for the present study and, if applicable, for the original study on which the present article is based  Page 26: We describe the funding sources for the study |
